# Regulation of Injury-Induced Neurogenesis by Nitric Oxide

**DOI:** 10.1155/2012/895659

**Published:** 2012-09-10

**Authors:** Bruno P. Carreira, Caetana M. Carvalho, Inês M. Araújo

**Affiliations:** ^1^Centre for Neuroscience and Cell Biology, Neuroendocrinology and Neurogenesis Group, University of Coimbra, Coimbra, Portugal; ^2^Regenerative Medicine Program, Department of Biomedical Sciences and Medicine, Gambelas Campus, University of Algarve, 8005-139 Faro, Portugal; ^3^IBB-Institute for Biotechnology and Bioengineering, Centre of Molecular and Structural Biomedicine, Gambelas Campus, University of Algarve, 8005-139 Faro, Portugal

## Abstract

The finding that neural stem cells (NSCs) are able to divide, migrate, and differentiate into several cellular types in the adult brain raised a new hope for restorative neurology. Nitric oxide (NO), a pleiotropic signaling molecule in the central nervous system (CNS), has been described to be able to modulate neurogenesis, acting as a pro- or antineurogenic agent. Some authors suggest that NO is a physiological inhibitor of neurogenesis, while others described NO to favor neurogenesis, particularly under inflammatory conditions. Thus, targeting the NO system may be a powerful strategy to control the formation of new neurons. However, the exact mechanisms by which NO regulates neural proliferation and differentiation are not yet completely clarified. In this paper we will discuss the potential interest of the modulation of the NO system for the treatment of neurodegenerative diseases or other pathological conditions that may affect the CNS.

## 1. Introduction

Neurogenesis is not limited to embryonic development as previously thought and occurs throughout the entire adult life of mammals, including humans. New neurons are continuously added to neural circuits and originate at two principal brain regions: the subventricular zone (SVZ) of the lateral ventricles, which generates olfactory bulb (OB) neurons, and the subgranular zone (SGZ) of the dentate gyrus (DG) of the hippocampus. Both regions harbor neural stem cells (NSCs) that can be isolated and cultured *in vitro* in the presence of growth factors, such as basic fibroblast growth factor (bFGF), epidermal growth factor (EGF), or both. The absence of growth factors results in the differentiation of cells into neurons, astrocytes, or oligodendrocytes as discussed in [1]. Neurogenesis has been exhaustively studied over the past years, and despite the great progress that has been achieved, the knowledge of the multiple aspects controlling proliferation, differentiation, or survival of NSCs is far from being known or understood. It was shown that neurogenesis decreases with aging and is impaired in several pathological conditions affecting the brain. Whether the insult is acute, such as ischemic brain stroke, traumatic brain injury, or epileptic seizures, or is a slow-progressing disease like Alzheimer's disease, Huntington's disease, or Parkinson's disease, all these conditions are accompanied by an inflammatory response in the brain [2]. Furthermore, the blockade of neuroinflammation restores adult neurogenesis [3, 4]. When an inflammatory response in the brain appears following an injury, activation of the brain immune cells takes place, particularly microglial cells. In inflammatory conditions, microglial cells become “activated”, and among a plethora of morphological and immunological alterations, they are able to express the inducible nitric oxide synthase (iNOS), producing high levels of nitric oxide (NO). 

NO is a multifaceted gaseous signaling molecule with several distinct functions in the central nervous system (CNS) [5]. This molecule is simultaneously involved in neuroprotection and in neurotoxicity, being also involved in inflammatory mechanisms in the CNS [6, 7]. NO was shown to modulate neurogenesis in the adult CNS as reviewed in [8]. In physiological conditions, NO tonically inhibits neurogenesis in the brain, while in pathophysiological conditions it exerts a proneurogenic effect on the dividing population of neuronal precursors. Moreover, the physiological effect of NO is mostly mediated by the neuronal nitric oxide synthase (nNOS), which is constitutively expressed, while pathophysiological levels of NO are attained following expression of iNOS [9–12]. Depending on the insult and on its source, NO can act as an antiproliferative agent [9–11] or stimulate neuronal precursor proliferation and differentiation [12]. However, the exact mechanisms by which NO regulates neuronal proliferation and differentiation are not yet clarified, and further investigation on this matter is needed. Since neuroinflammation is detrimental for adult neurogenesis, it would be of great interest to elucidate the role of inflammatory NO on the ongoing neurogenesis in these conditions. Therefore, the main goal of this paper is to elucidate the potential of the NO system modulation for the treatment of neurodegenerative diseases or other pathological conditions that may affect the CNS.

## 2. Neurogenesis following Brain Injury

Adult neurogenesis is implicated in many forms of plasticity in the CNS. The neurogenic process can be summarized in five main stages: (a) precursor cell proliferation, (b) fate determination, (c) migration, (d) differentiation and integration, and (e) survival.

Various models of injury in the rodent brain have been used to demonstrate that proliferation of stem cells is particularly enhanced in the SVZ and DG after an insult, which has been suggested to be a repair attempt from the lesioned brain, as reviewed in [13]. It has been observed that injury and pathological conditions affect adult neurogenesis, having a particular impact in neurogenic regions, but also in areas that are not normally considered as classical neurogenic regions, as discussed in [14, 15]. 

Regarding the type of insult to the brain, this may be acute, as ischemic brain stroke, traumatic brain injury or prolonged seizures, or a slow-progressing neurodegenerative disease. Neurogenesis decreases with aging and is impaired in several neurodegenerative disorders, such as Huntington's disease [16, 17] or Alzheimer's disease [18]. All these conditions are accompanied by an inflammatory response in the brain. However, the factors that attract neural progenitors to the lesioned areas are still under investigation. Another matter of hot debate is whether these new neurons are functionally integrated and survive in the existing neuronal circuitry.

## 3. Injury and Neuroinflammation

Inflammation is, by definition, a complex biological response to certain noxious stimuli such as stress, injury, or infection by external agents [19, 20]. After injury or exposure to pathogens, an inflammatory response takes place, with the involvement of two major groups of immune cells: (a) CNS resident microglial cells and astrocytes and (b) infiltrating lymphocytes, monocytes, and macrophages from the hematopoietic system [21, 22]. Therefore, the neuroinflammatory response attempts to protect the affected organism by removing harmful stimuli or removing dead and damaged cells, thereby initiating the healing process and return the tissue to homeostasis. When activated, immune cells release different regulating substances, such as complement molecules, cytokines-like interferon (IFN)-gamma, tumor necrosis factor (TNF)-alpha, interleukin (IL)-1beta, IL-18 and IL-6, chemokines such as stromal-derived factor (SDF)-1alpha and monocytes chemoattractant protein-1 (MCP-1), glutamate, reactive oxygen species (ROSs), and reactive nitrogen species (RNSs) like NO, as extensively reviewed in [23]. These inflammatory mediators are responsible for the recruitment of resident microglia, stimulation of astrogliosis, but also for the disruption of the blood-brain barrier (BBB) and further recruitment of monocytes and lymphocytes from the hematopoietic system to the site of inflammation [24–26].

Although inflammation in the CNS should be considered as a process that seeks to protect, we also must take into account its harmful properties as reported in [27]. The activation of recruited cells to the site of inflammation leads to the release of inflammatory factors that contribute to create a positive feedback loop of inflammatory activation, resulting ultimately in neuronal loss and/or neuronal damage. Thus, the inflammatory response may have a dual effect on the cellular environment, beneficial and/or detrimental. The severity of neuroinflammation can range from mild acute to uncontrolled chronic inflammation, resulting in different activation states of inflammatory cells and distinct biological outcomes [28]. It is believed that neuroinflammation may be involved in the mechanisms that lead to various CNS diseases, also affecting the process by which new neurons are generated in the brain [29].

### 3.1. Neurodegeneration

Neurodegeneration is characterized by the slow progressive dysfunction and loss of neurons in the CNS. Immune activation within the CNS is a classical event following infections, ischemia, trauma, and neurodegenerative diseases. The inflammatory response often contributes to collateral CNS injury, which is characterized essentially by neuronal loss and atrophy in different brain regions. Neuronal susceptibility to cell death [30, 31] and concomitant failure in self-repair mechanisms [32], combined with inhibition of axonal growth and limited repopulation by neuronal precursor cells are singled out as the main causes for neurodegenerative events that follow brain inflammation [33, 34]. However, not all immune response in the CNS should be considered harmful, and in many cases they actually are an important aid for cell repair and regeneration. Particularly, microglial cells seem to play an important role in facilitating the reorganization of neuronal circuits and in triggering repair [35]. Thus, like inflammation, microglial activation also appears to play a dual role in neurodegeneration, acting either as detrimental or beneficial, as reported in [36]. 

The relationship between neuroinflammation and neurodegeneration is being studied in numerous models of CNS disorders such as Alzheimer's and Parkinson's disease, suggesting neuroinflammation as a critical process, if not the primary cause, for CNS lesions seen in these diseases, as extensively reviewed in [23, 37]. However, these studies also revealed complex neuroimmune interactions, both at cellular and molecular levels, thus demonstrating that immune cells secrete both neurotoxic and neuroprotective molecules [2]. Although different triggering events could occur, a common feature for the neurodegenerative event seems to be the chronic activation of microglial cells.

### 3.2. Neuroinflammation and Production of New Neurons

As mentioned in previous sections, neuroinflammation is a complex process with different outcomes in neurogenesis, which can be enhanced or suppressed [38]. Besides differences between mild acute and uncontrolled chronic inflammation, the shift from pro- to antineurogenic inflammatory status appears to be dependent on (a) the mechanism by which microglia, macrophages, and/or astrocytes are activated, (b) the type of inflammatory mediators released, and (c) for how long inflammatory cells, particularly microglia, are activated [36]. 

#### 3.2.1. Impaired Formation of New Neurons

Inflammation and microglia activation were initially thought to inhibit adult neurogenesis [3, 4], while recent studies indicate that microglia can also support neurogenic events, as described in [39]. It was shown that lipopolysaccharide-(LPS-) induced activation of microglia impairs neurogenesis in rats [4], apparently through the increased production of TNF-alpha [40]. Additional evidences corroborating the detrimental effect of LPS-activated microglia was provided by another study, which showed that acute activation of microglia with LPS reduces NSC survival and neuronal differentiation [41]. Furthermore, suppression of microglial activation with an antibiotic, such as minocycline, was also used to demonstrate increased neurogenesis in the hippocampus, thus indicating that the severity of impaired neurogenesis correlates with the number of activated microglial cells [4]. Several other authors reported that the mechanism by which microglia exert these effects involves the release of proinflammatory mediators, such as IL-1, IL-6, IFN-gamma, and TNF-alpha, which seem to play an essential role in suppressing neurogenesis [42–45] (Table 1). It has also been suggested that ROS and RNS, particularly NO, can inhibit adult neurogenesis in inflammatory conditions [3, 46, 47]. In addition, several studies demonstrated that neurogenesis could be restored following treatment with anti-inflammatory drugs [3, 4, 48, 49]. Neurogenesis was restored after treatment with indomethacin, a nonsteroidal anti-inflammatory drug (NSAID), after irradiation-induced inflammation [3] or focal cerebral ischemia [49]. Other studies also reported an increased survival of newly generated neuroblasts in the striatum after stroke [49], or in the DG after middle cerebral artery occlusion (MCAO) [50] when the activation of microglia is inhibited by indomethacin or minocycline, respectively.

#### 3.2.2. Enhancement of Neurogenesis

Contrary to what was initially thought that neuroinflammation is detrimental to adult neurogenesis, recent evidence indicates that under certain circumstances inflammation can also benefit the neurogenic process (Table 1). Apparently, neural stem cells become “activated” following brain injury and migrate into the lesioned areas, thus suggesting the inflammatory microenvironment as an important trigger for the migration of newborn cells [64, 65]. Microglia was reported to play a dual role on neurogenesis, suggesting neurogenesis inhibition to be caused by microglial activation under inflammatory conditions [53]. Other studies showed a persistent production of neurons from adult NSC, even after the inhibition of acute microglial activation, during recovery after stroke [66, 67]. Moreover, it was demonstrated that long-term survival of newborn neurons after *status epilepticus* (SE), with concomitant chronic activation of microglia [68]. *In vitro* studies have also showed an important role for microglia in directing the replacement of damaged or lost cells [52, 69–71]. LPS-activated microglia and inflammation increase the integration of newly generated neurons into the adult rat hippocampus [72]. More recently, long-term accumulation of activated microglia, although with a downregulated inflammatory profile, was shown to be concomitant with persistent neurogenesis in the adult SVZ after stroke [73]. Other inflammatory mediators have also been implicated in the improvement of migration and proliferation of new neurons following brain damage, such as SDF-1alpha and its receptor CXCR4 [62, 66] or trophic factors such as GDNF and BDNF, who are involved in the removal of damaged synapses [73]. In summary, all these studies suggest a neuroprotective role of microglia for newborns cells. Although microglia may have a detrimental action in early stages of the inflammatory response that follows acute insults, it could be converted into a protective state during chronic activation. 

#### 3.2.3. Dual Role of Inflammation in Neurogenesis

It is now widely accepted that microglia have a dual role in neurogenesis by favoring it or, alternatively, hindering neurogenesis. Apparently, microglial cells and the inflammatory factors they release, like NO (to be discussed below), seem to have opposite roles in neurogenesis under inflammatory conditions [38, 74]. However, it is important to reinforce the idea that inflammation, essentially characterized by activation of microglia, has distinct roles in various stages of neurogenesis, this effect being dependent on the degree of activation of immune cells, type of inflammatory mediator released, and duration of the inflammatory response [38]. Nevertheless, there are lines of evidence for some of the most important inflammatory mediators in the regulation of neurogenesis and/or neuroprotection [23, 75, 76].

As noted, further studies should be conducted to assess the interaction between neuroinflammation and neurogenesis, particularly how neuroinflammation modulates self-renewal, proliferation, migration, differentiation, integration in the neuronal network, and, more importantly, survival of newborn cells. As different authors have reported that chronic inflammation can stimulate one or more stages of neurogenesis, such as migration, proliferation, or differentiation, the problem remains in the reduced long-term survival of newborn neurons [23]. Moreover, since different microglial phenotypes and morphologies can be identified during inflammation, an extensive genetic and proteomic characterization will be of great interest to understand more accurately this complex crosstalk. 

## 4. Nitric Oxide

Nitric oxide, a short-lived gaseous-free radical, is synthesized by the nitric oxide synthase (NOS) family of enzymes present in most of the cells of the body. NO is implicated in a wide range of physiological processes within the cardiovascular, immune, and nervous system, where it can act as a non-canonical neurotransmitter [77], but it can also be an important player in pathophysiological events. Different members of the NOS family control different functions of NO. The discovery of NO in the CNS was a breakthrough in the concept of neuronal communication. NO was characterized in the CNS for the first time as an intracellular messenger to increase cyclic guanosine 3′,5′-monophosphate (cGMP) levels, after the activation of glutamate receptors [78]. Later, the same authors also described NO as a neuromodulator, particularly due to its diffusible properties [79], thus acting not only in cells that release NO, but also in neighboring cells where it can therefore trigger its autocrine and/or paracrine functions. Unlike other neurotransmitters, NO is synthesized on demand, diffusing from nerve terminals since it is not stored in vesicles nor released by exocytosis [5]. In the CNS, NO is also associated with cognitive function, having an important role in synaptic plasticity, and controls biological functions, including body temperature, sleep-wake cycle, appetite, and modulation of hormone release, as reviewed in [7]. Another distinctive feature from classical neurotransmitters is that, unlike them, NO ends its action after reacting with a substrate and not by enzymatic degradation or reuptake. In addition, the key mechanism to regulate the activity of NO is the control of its synthesis. 

Physiologically, NO interacts with several intracellular targets activating different signaling pathways with a stimulatory or inhibitory response. However, NO can also be toxic to cells, in a mechanism dependent on the formation of RNS [80, 81]. Oxidative stress and nitrosative stress, a consequence of high levels of NO and RNS, have been implicated in the pathogenesis of several neurodegenerative disorders [80, 82, 83], which will be explored in Section 4.2.

### 4.1. NO as an Inflammatory Mediator

The NOS family of enzymes is responsible for the synthesis of NO. Three different enzyme isoforms have been identified in mammalian cells: (a) neuronal NOS (nNOS, type I), which is constitutively expressed in brain neurons and is activated by calcium/calmodulin, particularly following stimulation of NMDA-type glutamate receptors; (b) endothelial NOS (eNOS, type III), constitutively expressed in endothelial cells and astrocytes and is regulated by calcium/calmodulin and phosphorylation/dephosphorylation; (c) inducible NOS (iNOS, type II) which is calcium-independent and its regulation depends on de novo synthesis [80, 84, 85]. iNOS is not normally expressed in the “healthy” brain but is induced in glial and endothelial cells by proinflammatory stimuli such as cytokines, bacterial/viral agents, and/or hypoxia [80]. iNOS is mainly expressed in macrophages, astrocytes, and microglial cells, upon neurotoxic, traumatic, and inflammatory damage [7, 84, 86, 87], but it could also be found in neurons [88, 89]. Once expressed, iNOS continuously produces high amounts of NO, even for several days [31, 87, 90–92]. The massive production of NO by iNOS is toxic, since it inactivates the mitochondrial respiratory chain enzymes that ultimately induce apoptosis in target cells. Moreover, NO has been described as an important activator of cyclooxygenase-II (COX-2) in glial cells, also regulating leukocyte adhesion in vessels [80]. The concentration achieved by NO seems to be a determining factor for the effects observed locally in the brain. Thus, in physiological concentrations, which are believed to range from 0.1 to 100 nM, NO is relatively nonreactive, and its actions are mainly mediated by binding to the heme group of soluble guanylate cyclase (sGC), leading to its activation and subsequent production of cGMP [93]. 

NO can also be converted into more reactive species commonly refereed as RNS. In high concentrations, NO reacts directly with oxygen (O_2_) to produce nitrogen dioxide (NO_2_), which in turn further reacts with NO originating dinitrogen trioxide (N_2_O_3_). In addition, NO_2_ may oxidize or nitrate, by adding a nitro (NO_2_^+^) group to a great variety of molecules, being a classic example the nitration of tyrosine to 3-nitrotyrosine [94]. Moreover, NO reacts with superoxide (O_2_^−^) to produce peroxynitrite (ONOO^−^), an extremely reactive molecule which can oxidize or nitrate other molecules or, instead, decay forming other damaging species, such as NO_2_ and/or the hydroxyl radical (OH^•^). On the other hand, N_2_O_3_ can add a nitrosonium ion (NO^+^) to thiols or amines, an event also designated as nitrosation/nitrosylation, being a good example cysteine than can be nitrosated to S-nitrosocysteine [94]. Both S-nitrosylation and nitration typically lead to alterations in protein function [94]. 

### 4.2. Neuronal Death

According to the literature, the role of NO in the brain could be summed up in two radically different outcomes: (a) as an intracellular signaling messenger, regulating a wide variety of physiological events, such as synaptic plasticity, blood flow, and neuronal development [95] and (b) as a cytotoxic agent killing indiscriminately both pathogenic and “healthy” host cells in disease [96, 97]. Strong evidence has been reported in the literature supporting a role of NO in the pathogenesis of neurodegenerative disorders, including autoimmune and chronic neurodegenerative diseases. As stated in previous sections, the role of NO seems to be dependent on the concentration attained locally in tissues. When produced in excess, NO shifts from a physiological to a neurotoxic effector. NO overproduction may be due to nNOS activation following persistent glutamate excitatory input and/or iNOS expression, upon an inflammatory response. Activated inflammatory cells generate increased levels of ROS such as superoxide, hydrogen peroxide, and hydroxyl radical. Moreover, NO can also induce the production of superoxide by mitochondria [7]. NO and superoxide readily react to form ONOO^−^, an extremely reactive molecule [81].

Likewise, the excessive release of both glutamate and NO, coupled to oxidative stress and mitochondrial dysfunction, appears to be involved in the majority of neurodegenerative diseases. NO from inflammatory origin has been reported as an important contributing factor to the vulnerability of neurons, causing neuronal death both *in vivo* and *in vitro* in rodents [98, 99]. Some authors have suggested this neurotoxic effect as a consequence of enzymatic inhibition of the respiratory chain, resulting in hypoxia, excitotoxicity, and elevated levels of ONOO^−^, as reviewed in [81]. Furthermore, the excessive NO release by glial cells leads to the formation of ONOO^−^, which appears to be involved in the mechanisms of neuronal death, some of them linked to protein dysfunction due to nitration or s-nitrosylation [100]. Protein nitration is an irreversible chemical modification affecting tyrosine phosphorylation or dephosphorylation, which seriously affects several signaling pathways involved in the control of cell survival, proliferation, or programmed cell death, as reviewed in [101]. 

Although it has been implicated in acute injury events, particularly due to a massive release during an inflammatory response, NO has also been associated to slow progressive disorders that can be genetically inherited or sporadic. Parkinson's disease, Alzheimer's disease, Huntington's disease, multiple sclerosis, and amyotrophic lateral sclerosis are all neurodegenerative disorders in which NO has been suggested to be involved, since all of them show evidence of oxidative and nitrosative stress [80, 102]. ROS and RNS are important factors in neuroinflammation-mediated neurotoxicity [103]. Furthermore, the presence of 3-nitrotyrosine has been reported in several neurodegenerative diseases linked to oxidative stress such as Alzheimer's [104] or Parkinson's disease [105]. Thus, understanding the involvement of NO in the etiology of these disorders may highlight an eventual beneficial potential role of selective NOS inhibitors.

### 4.3. Nitric Oxide and Neurogenesis

The role of NO as a modulator of neurogenesis is a matter of strong debate. Depending on the source, NO has a dual influence in the neurogenic process both by inhibiting or stimulating neurogenesis (Table 2).

The role of NO in neurogenesis has not been identified until recently [9, 10, 110]. The authors of these contributions had also described a cytostatic function of NO in the CNS, demonstrating that nNOS-derived NO is involved in the regulation of neurogenesis, particularly neural stem cell function [9, 10, 110]. Since blood vessels are part of the SVZ and dentate gyrus SGZ niches, which are also surrounded by differentiated neurons expressing nNOS, NO is produced in close proximity to NSCs. Several authors have described another function for NO in the rostral migratory stream (RMS), where SVZ-derived progenitor cells migrate into the olfactory bulb and differentiate into neurons [119]. These authors demonstrated that nitrergic neurons are in close vicinity to the RMS and that the NO generated regulates the migration and proliferation of progenitors that could also express nNOS [119]. Other groups have demonstrated NO production to be induced by neurotrophic factors, which in turn act in target cells inducing cell cycle arrest and/or exit favoring differentiation [111, 120, 121].

It should be noted here that the majority of the studies on the effect of NO in adult neurogenesis are focused mainly on the modulation of proliferation. In this context, the evaluation of survival rates of newly formed neurons is also important, since NO is known to be a regulator of apoptosis [118]. Several studies have shown that NO inhibits apoptosis by preventing increases in caspase-3 activity [122], which has been described to increase short-term survival of progenitor-cell progeny in the adult rat DG following SE [123]. 

Production of NO via nNOS has been demonstrated to have an important antiproliferative effect both *in vitro* and *in vivo*, but also as being involved in neuronal differentiation, survival, and synaptic plasticity [9, 10, 107, 113, 124]. It was shown that chronic nNOS inhibition enhances neurogenesis. Indeed, the selective inhibition of nNOS with 7-nitroindazole (7-NI) greatly increased cell proliferation in the SVZ, RMS, and OB, but not in the DG, in adult mice [10]. This antiproliferative effect of NO has been confirmed by others, that have shown that when NO production is inhibited either by using an intraventricular infusion of an NOS inhibitor in the rat brain or by using an nNOS-knockout mouse model, proliferation is greatly increased in the olfactory subependymal zone and in the DG [9, 108, 113, 125]. Moreover, the inhibitory role of nNOS-derived NO on SVZ and DG neurogenesis has also been demonstrated in the context of cerebral ischemia [126]. Other authors suggested NO to be a negative regulator of SVZ neurogenesis by modulating the activity of the EGF receptor [107], via nitrosylation of specific cysteine residues [127] (Table 3). Accordingly to these studies, the antiproliferative effect can be partially explained by the inhibition of the EGF receptor and the phosphoinositide-3-kinase (PI3-K)/Akt signaling pathway [107, 127]. Moreover, these authors described the antimitotic effect of NO to correlate with the nuclear presence of the cyclin-dependent kinase inhibitor p27^Kip1^ [127]. 

On the contrary, by using pharmacological or genetic approaches, an opposite role has been found for NO synthesized by eNOS in the SVZ and iNOS in the DG following focal ischemia, which seems to stimulate neurogenesis [12, 128]. Moreover, increased immunoreactivity against iNOS following transient ischemia was shown to correlate with a decrease of nNOS in the hippocampus, which is concomitant with an increased neurogenesis [116, 129]. Numerous works showed that ischemia-induced neurogenesis in DG involves the activation of NMDA receptors [130], which is simultaneous to increased iNOS expression [131, 132] (Table 3). However, in a study regarding the effects of NO in cell proliferation, both nNOS- and iNOS-derived NO increases neurogenesis following seizures in the DG of adult rats [133]. Other authors reported that NO released under inflammatory conditions is involved in NSC differentiation into astrocytes by a mechanism dependent on the activation of the JAK/STAT-1 signal transduction pathway [112]. Recently we showed that supraphysiological levels of NO induce the proliferation of SVZ-derived neural stem cells through the activation of two signaling pathways, in a biphasic manner. Thus, the mitotic effect of NO is initially mediated by the direct activation of signaling pathway downstream of the EGF receptor, but bypassing the EGF receptor [74]. Downstream of the EGF receptor, there is an increased activation of the mitogen-activated protein (MAP) kinase ERK pathway following exposure to NO, which activates several downstream targets, namely p90RSK, and further decreases nuclear levels of p27^Kip1^, thus allowing cell cycle progression [74]. Furthermore, the proliferative effect of supraphysiological levels of NO, following longer periods of exposure (24 h), is mediated by increased signaling through the cGMP/cGMP-dependent kinase (PKG) pathway [115]. In addition, we also showed that NO from iNOS origin promotes proliferation of NSC in the hippocampus of adult mice following SE [74].

Altogether these findings illustrate that NO is a modulator of neurogenesis in diverse ways, and the different NO synthases are important players in this effect on neurogenesis [11, 139, 140]. NO effects on neurogenesis are dependent on the developmental period and source of NO (Table 3). Furthermore, NO can have concentration-dependent effects, depending on the local concentration and surrounding molecular environment. Apparently, under physiological conditions NO acts as a negative regulator of neurogenesis [9, 10, 110], while in inflammatory conditions a decrease in nNOS and increase in iNOS may act as a mechanism to enhance neurogenesis [12, 74, 107, 141, 142]. However, the exact molecular mechanisms underlying this dual effect of NO on neurogenesis, are not fully clarified and more studies need to be conducted.

## 5. Potential Neurogenic Targets in Nitrergic Pathways

Repair of damaged tissues and organs is essential for the survival of organisms. Although the CNS has pools of neural stem cells, these have a limited ability for repair and endogenous cell replacement. Some strategies have been studied over the past years to promote brain repair, particularly: (a) neural precursor or stem cell transplantation or (b) stimulation of endogenous neurogenesis. Moreover, excessive proliferation of NSCs associated with tumor formation is a major concern in the clinical application of both these strategies. Since most brain disorders that could benefit from enhanced neurogenesis are normally accompanied by neuroinflammation, understanding how the inflammatory response affects the neurogenic process is of major importance for the design of safe and efficient therapeutic strategies.

As discussed previously, NO was described to have a dual role on the regulation of adult neurogenesis. NO synthesized from nNOS appears to decrease neurogenesis or to act as an antiproliferative agent [9, 10, 107, 108, 110, 113, 127], whereas NO from iNOS and eNOS origin seems to stimulate neurogenesis [12, 74, 128, 142]. Taking this evidence into account, the modulation of the NO system may be a good target for the development of strategies to improve brain repair. Next, some of the most relevant therapeutic strategies for brain repair using the modulation of the nitrergic system will be discussed.

### 5.1. Nitric Oxide-Releasing Drugs

Nitric oxide-releasing drugs are pharmacologically active substances that release NO *in vivo* or *in vitro*. Two large groups of NO-releasing drugs can be found today: (a) NO donors and (b) NO-releasing nonsteroidal anti-inflammatory drugs. Although the clinical application of these drugs to improve brain repair seems remote, their potential application in the treatment of CNS disorders is a matter of great interest. Several studies have been carried out in order to understand how these drugs control neurogenesis. In fact, there seem to exist good reasons to believe that the use of these drugs may be advantageous in the treatment of brain disorders.

#### 5.1.1. Nitric Oxide Donors

Nitric oxide-releasing compounds are clinically used for the treatment of patients with coronary heart disease [143]. Different types of NO-releasing agents have been developed and are commercially available, such as sodium nitroprusside (SNP), firstly described as a vasodilator, which is used to manage acute hypertensive crisis; or molsidomine, used in the therapy of angina pectoris and heart failure. SIN-1, another NO donor, is known as both NO and ONOO^−^ donor mainly because during NO release from SIN-1 superoxide is also generated [144, 145]. A wide range of NO-releasing drug classes have been developed recently. Among them are diazeniumdiolates, also known as NONOates (such as DEA/NO, SPER/NO, or DETA/NO) that release NO spontaneously under physiological conditions. Preclinical studies have shown a potential application for NONOate in cardiovascular disease, but further studies need to be conducted for their use in the clinic [145]. Chemically distinct NO donors differ in their half-life time and amounts of NO released *in vitro*. Moreover, depending on pH value, temperature, presence of cofactors, and light, the amount of NO released could be altered [144–146]. 

These compounds have also been useful to study physiological processes and molecular mechanisms in which NO is involved. NO was described to act as an antiproliferative agent in the CNS under physiological conditions, thus affecting neurogenesis [127]. Interestingly, in this work the authors described NO to be antiproliferative through the inhibition of the EGF receptor by S-nitrosylation [127]. Moreover, other authors have also described NO physiological levels to be antiproliferative in the brain [11, 107]. 

Numerous studies have used NO donors to investigate the effect of high concentrations of NO on neurogenesis, thus mimicking NO concentrations that can be achieved locally in the brain following an inflammatory response. Several groups reported nitric oxide-releasing drugs to enhance recovery after brain injury, partly by increasing neurogenesis in the DG and SVZ [147–150], following ischemic stroke [147, 151] and traumatic brain injury [148]. One study found that exogenous administration of NO using DETA/NO increases cell proliferation and survival in mice hippocampus [117]. We have shown that high concentrations of NO, which could be attained locally in the brain following an inflammatory response, have a dual effect on the proliferation of SVZ-derived NSCs [74, 115]. In fact, the effect of NO on the proliferation appears to be dependent on the period of exposure and concentration of NO achieved. Thus, a slight elevation on NO levels above the physiological range has a proliferative effect in an initial stage (1 day). On the contrary, continuous release of NO overtime (for 2 days) had an antiproliferative effect in SVZ-derived NSCs [74, 115]. This evidence is important to realize that controlling neuroinflammation, thus controlling NO production, will improve the outcome from the neurogenic process following brain injury. Other groups have shown that high concentrations of NO could also modulate other neurogenic stages, such as migration [136, 152] or differentiation [112]. 

In fact, the studies published in the literature about the effect of high levels of NO in neurogenesis, using NO donors, seem to bring contradictory evidence. However, it should be noted that in most of these works the NO donors used are chemically distinct and/or have distinct kinetics on NO release. Therefore, the evidence should be carefully interpreted to prevent misleading conclusions. Nevertheless, all these studies appear to be consensual on the following: NO is an important modulator of neurogenesis.

#### 5.1.2. Nitric Oxide-Releasing Nonsteroidal Anti-Inflammatory Drugs

Nitric oxide-releasing nonsteroidal anti-inflammatory drugs (NO-NSAIDs) are a group of compounds with potential therapeutic applications in several clinical conditions. These drugs are synthesized by grafting a NO-donating moiety to classical NSAID, such as aspirin (NO-aspirin), flurbiprofen (NO-flurbiprofen), naproxen (NO-naproxen), diclofenac (NO-diclofenac), and ibuprofen (NO-ibuprofen) [143, 153, 154]. At present, NSAIDs are used for the treatment of a variety of inflammatory conditions. However, NSAIDs have a limited therapeutic application in chronic conditions, mainly due to their significant side effects in the gastrointestinal (GI) tract and kidneys. In the last decades, a great effort has been done to improve NSAID safety. Therefore, NO-NSAID may be considered as an important therapeutic attempt to overcome the side effects by NSAID. The release of NO from these drugs mimics the physiological production of NO by constitutive NOS, which appears to reduce the toxicity when compared to the parent NSAID [153, 155]. Moreover, this modification strongly reduces the side effects of NSAID, without affecting the anti-inflammatory effectiveness [153].

Since NSAIDs are primarily used as anti-inflammatory drugs, many of the studies with NO-NSAID have been essentially about its anti-inflammatory effects. Numerous studies in the literature have reported the anti-inflammatory effect of NO-NSAID in animal models of acute or chronic inflammation. More recently, there has been increasing concern about the potential application of these drugs in CNS disorders, particularly in neurodegenerative diseases, such as Alzheimer's disease. Numerous reports suggested NO-NSAID to be a suitable approach for the treatment of Alzheimer's disease, since they are less toxic to the GI tract than NSAID following chronic ingestion. Moreover, NO-NSAID also inhibit caspase activity thus protecting neurons against cytokine-induced apoptosis during Alzheimer's disease [153]. As reported by Hauss-Wegrzyniak and coworkers, chronic ingestion of NO-flurbiprofen reduced the activity state of microglial cells in a rat model of Alzheimer's disease, when comparing to animals treated with aspirin [156]. Other authors also described NO-flurbiprofen to reduce brain beta-amyloid in a mice model of Alzheimer's disease, which was associated with activation of microglial cells, the presumed responsible for clearing beta-amyloid deposits [157]. Interestingly, these authors reported NO-aspirin to be more efficacious than ibuprofen or celecoxib, a selective COX-2 inhibitor [157]. The neuroprotective effect of different NO-NSAID has additionally been described in other animal models of brain damage. Treatment with NO-aspirin was shown to be more neuroprotective than aspirin, following MCAO [158]. In fact, the results from these experiments are of great interest since they strongly suggest that NO release is determinant for the protective action of NO-aspirin in this animal model. Although the mechanism underlying this effect is still unclear, NO improved blood flow to the ischemic region, thereby reducing the lesioned area. Moreover, the ability of NO-NSAID to inhibit caspase activity is also important for this effect [159].

Given the ability of NSAID in crossing the BBB [160], the use of NO-NSAID in the treatment of CNS disorders can be a very useful tool, in particular for the control of neuroinflammation that, as noted above, may affect neurogenesis [155]. Therefore, it is important to conduct more studies to understand the mechanisms and levels within which NO released by NO-NSAID may promote neurogenesis.

### 5.2. PDE Inhibitors

The main cellular signaling pathway stimulated by NO is the activation of sGC, subsequent production of cGMP, and further activation of protein kinases that regulate various physiological events [161]. Neurons synthesize cGMP in response to NO by activation of sGC, a heterodimeric heme-containing enzyme. NO reacts with the heme group of the sGC, which undergoes a conformational change, converting GTP into the second messenger cGMP [93, 162]. Some studies suggest that NO can also downregulate sGC activity, particularly in neuroinflammatory conditions [163]. cGMP-dependent kinases, which are serine/threonine kinases, are activated by cGMP and are involved in several physiological phenomena including long-term potentiation in the hippocampus and long-term depression in the cerebellum [93, 162]. In physiological conditions, intracellular cGMP levels are controlled by cyclic nucleotide phosphodiesterases (PDEs) [94]. PDEs are enzymes that hydrolyze the 3′-phosphodiester bound of cyclic adenosine monophosphate (cAMP) or cGMP, originating their corresponding monophosphates, 5′-AMP or 5′-GMP, respectively. cGMP-related physiological functions can be regulated by controlling the levels of PDE type 5 (PDE5) enzymes, which specifically hydrolyze cGMP. Moreover, cGMP also modulates the activity of PDE [164]. 

The use of selective PDE inhibitors has been proven to be useful in the clinic, particularly PDE5 inhibitors, which are drugs used to treat erectile dysfunction and pulmonary arterial hypertension [164–166]. Sildenafil, commercially available as Viagra, is classically considered as a PDE5 inhibitor; however, it also inhibits PDE1 and PDE6 [166–168]. Similarly to sildenafil, two other inhibitors with higher selectivity for PDE5 were developed for the treatment of erectile dysfunction: tadalafil (Cialis) and vardenafil (Levitra). More recently, a new compound was developed, T0156, which potently inhibits PDE5 [169]. In fact, T0156 inhibits PDE5 with higher potency than sildenafil also presenting higher selectivity for PDE5 in comparison to PDE6 [169]. In erectile dysfunction, PDE5 inhibition enhances relaxation of the cavernosal smooth muscle by NO and cGMP, thus allowing blood flow and stimulating penile erection [170, 171]. In the lung, PDE5 inhibitors act as vasodilators, increasing blood supply, antagonizing the vasoconstriction of smooth muscle, and decreasing pulmonary arterial resistance, thus treating pulmonary hypertension (for comprehensive review see [172–174]). 

In the CNS, neurogenesis generally declines with aging and is correlated with the emergence of neurodegenerative diseases. Moreover, the levels of NO gradually decrease in aging, which is concomitant with a decrease in cGMP levels. As demonstrated in aged rats, cGMP levels are decreased as a consequence of the increasing phosphodiesterase activity when compared to young adult rats [175]. Several authors described NO and cGMP to be important effectors in the regulation of different events related with the neurogenic process, particularly proliferation, migration, differentiation, growth, axon guidance, and cell survival [115, 136, 152, 176, 177]. Furthermore, brain PDE5 was reported to have a role in learning and memory, physiological events that are closely dependent on neurogenesis. Therefore, targeting PDE5 activity as a strategy to reverse the deleterious effects on neurogenesis, and thus enhancing it, seems to be a promising strategy to be applied in clinic. However it should be noted that the use of PDE5 inhibitors as an effective therapy for neurodegenerative diseases is dependent on their permeability to the BBB. For instance, sildenafil is known to cross the BBB and can be easily administered. 

The administration of PDE5 inhibitors as a possible therapy for Alzheimer's disease has been studied, due to their ability to reverse deficits in long-term memory caused by pharmacological agents or aging. Different authors have described that the administration of sildenafil enhances memory and restores learning ability in animal models [178–184]. Beyond this important role, PDE5 inhibitors appear to stimulate neuronal plasticity, particularly through the enhancement of endogenous neurogenesis in the adult brain. In addition, the administration of PDE5 inhibitors, such as sildenafil, but also tadalafil, positively affected neurogenesis in the OB, SVZ, and the DG of rats by a mechanism involving the intracellular increase of cGMP levels [185–187]. Moreover, the administration of PDE5 inhibitors has also been associated to neuronal function recovery in rats following a stroke [188] or after ischemic injury either in young adult rats as in aged rats [187, 189]. Furthermore, PDE5 inhibition by sildenafil stimulated cell proliferation in rat SVZ cultures [190]. In a recent report, it was shown that sildenafil has a neuroprotective role, improving the clinical symptoms and neuropathology in a mouse model of multiple sclerosis, thus suggesting PDE5 as an important target for the therapy of this disease [151]. 

In summary, this evidence supports the idea that the use of PDE5 inhibitors merits further investigation in order to clarify their involvement on neurogenesis, but also to understand the mechanisms underlying these effects.

## 6. Future Prospects

Stimulation of endogenous adult neurogenesis and modulation of injury-induced neurogenesis is presently being considered as a potential therapeutic approach for neuronal repair in neurodegenerative disorders, as opposed to the more invasive approach of transplantation of exogenous stem cells. Understanding how the inflammatory response affects neurogenesis is fundamental to better design therapeutic strategies for safe and efficient regulation of endogenous neurogenesis. Therefore, the knowledge of the inflammatory agents that modulate proliferation and/or differentiation of NSCs is of great usefulness if its action could be correctly targeted and controlled, for instance, with selective drugs for the agent of interest. 

Nitric oxide, which acts as a nonspecific cytotoxic mediator and a biological messenger, in immunological response, has been attracting increasing importance from pharmaceutical companies. Indeed, several nonsteroidal anti-inflammatory NO-releasing drugs (NO-NSAID) are currently under investigation and were shown to be beneficial in models of several neurodegenerative conditions accompanied by inflammation [153, 191]. As an alternative to conventional NSAIDs with significant side effects, pharmacologically improved and therapeutically enhanced NO releasing nonsteroidal anti-inflammatory drugs with less side effects are being developed as reviewed in [192]. Moreover, besides the clinical applications of PDE5 inhibitors, they appear to be a good strategy for the treatment of certain CNS disorders and further improve neurogenesis. These drugs have already been shown to be important modulators of the nitrergic system, preventing neurodegeneration and favoring neurogenesis. 

In light of these facts, the modulation of the NO system seems to be a good target for the development of strategies to improve brain repair. However, despite all good evidence that drugs that modulate the NO system have given, further studies are necessary. In fact, a full understanding of how inflammation affects neurogenesis is essential to the development of therapeutic strategies that can induce neurogenesis from endogenous neural precursor cells, and further investigation needs to be conducted to better understand the mechanisms underlying the effect of neuroinflammation in cellular regeneration in the diseased brain.

## Figures and Tables

**Table 1:** Regulation of adult neurogenesis by inflammatory mediators.

Inflammatory factor	Proliferation of NSC	Differentiation of NCS	Survival of NSC	References
IL-1	↑ or ↓	—	↓	[51]
IL-6	↓	↓ neuronal	↓	[4, 46, 52]
IFN-gamma	↓	↑ neuronal	↓	[42, 53–56]
	↑	—	=	[57, 58]
TNF-alpha	—	↓ neuronal	↓	[40]
	—	—	↓	[43, 54]
	↑	↓ neuronal (TNF-R1)	=	[59]
	↑	↑ neuronal (TNF-R2)	↑	[60]
	↑	↑ neuronal (TNF-R1)	↑	[61]
		↑ astrocytic		
	↑ or ↓	↑ neuronal (TNF-R2)	↑ or ↓	[36, 44]
		↓ neuronal (TNF-R1)		
SDF-1alpha	↑ or ↓	↑ neuronal	↑	[62, 63]

The effects listed here may not be direct. ↑: increase; ↓: decrease; =: no change; —: no report.

**Table 2:** Regulation of adult neurogenesis by NO under physiological or inflammatory conditions.

Condition	Proliferation of NSC	Differentiation of NCS	Survival of NSC	References
Physiological	↓	=	=	[8, 9, 106, 107]
	↓	=	↓	[8, 108, 109]
	↓	↑	=	[8, 10, 11, 110–112]
	↓	↓	↓	[8, 113, 114]

Inflammation	↑	=	=	[23, 115]
	↑	↑	=	[12, 23, 74, 116, 117]
	↑	↑	↓	[23, 118]

The effects listed here may not be direct. ↑: increase; ↓, decrease; =, no change or no report.

**Table 3:** NO-dependent signal pathways in neurogenesis.

NO source	Effect	Signaling pathway	References
nNOS	↓ proliferation (SVZ)	Nitrosylation of EGF receptor	[127]
		(PI3-K)/Akt pathway	[107, 127]
	↓ neurogenesis (DG)	PSA-NCAM and CREB	[134]
		cAMP phosphorylation	[113]
eNOS	↑ neurogenesis (DG and SVZ)	↑ BDNF and VEGF	[135]
	↑ neurogenesis (DG)	↑ VEGF	[128]
iNOS	↑ proliferation (SVZ)	ERK 1/2 pathway	[74]
		cGMP/PKG pathway	[115]
	↑ migration (NT2 cell line)	cGMP/PKG pathway	[136]
	↑ neurogenesis (DG)	NMDA receptor	[130, 137]
	↑ neurogenesis (DG and SVZ)	L-VGCC	[138]
	↑ astrogliogenesis	JAK/STAT-1 pathway	[112]

↑: Increase; ↓: decrease; Brain-derived neurotrophic factor, BDNF; Vascular endothelial growth factor, VEGF; L-type voltage-gated Ca^2+^ channel, L-VGCC.
